# Topics of Public Interest

**Published:** 1915-05

**Authors:** 


### TOPICS OF PUBLIC INTEREST.

---

**Automobile Statistics.** In 1901, 954 owners' licenses were issued in the state of New York. In 1914, 169,966 owners' and 66,636 chauffeurs' licenses were issued. At the rate of increase for the last few years, just about 200,000 cars will be in use in the state this year, about one for every ten average families. Will you be decent enough to give rides, during the season, to this number of persons? Such a courtesy does not involve any appreciable expense or loss of time, just a little thoughtfulness, a little sacrifice of privacy and pride, a slight loss of speed when you pass a woman with a bundle or a man with a suit case on a country road. The courtesy will pay in the long run for it will give pedestrians and drivers of horses a better feeling toward drivers of automobiles, a better understanding of the difficulties of avoiding accidents and delays on highways and, indirectly it will tend toward better roads and lower special taxes.

---

**Graded Milk.** Beginning April 1, the state law came into practical operation, under the supervision of Stephen W. Bateson, acting chief of the bureau of food and drugs. All farms supplying milk to cities must be inspected and rated as to equipment and methods. The bottle caps will designate three grades: A. tuberculin tested cows, certified and registered, minimum score of 25 per cent. on equipment and 50 per cent. on methods at farm, delivery to customer within 36 hours; B. gross inspection and registry of cows, 23 per cent. and 37 per cent. scores, respectively, same limit as to delivery. C. same as B. except total score required 40 per cent. delivery limit 48 hours. Over 2,600 farms supply milk for Buffalo. These restrictions are not an unmixed blessing: they tend to produce confidence which will fail to be realized in individual cases, they will drive small dealers out of business and will inevitably increase prices.

---

**Medical Bills** reported adversely by the Public Health Committee of the New York Assembly: Tallett's to regulate nursing—too arbitrary and too restrictive of term **nurse**; Tallett's to regulate and legalize chiropractice—objections obvious; Seelye's to provide for registration of dentists; Seelye's to disbar advertising physicians—contrary to general principle that the law should confine itself to gross crimes and damages to person and property and should not invade the domains of pure morality and ethics, also objectionable as it could not deal effectively with sneaking methods of publicity and might potentially be abused on personal grounds, as it would give judicial powers in each case to the Regents. In reference to the last bill, while the active opposition was mainly from advertising physicians, the profession generally seems to feel that there ought to be something left to personal conscience, gentlemanly instincts, etc. Laws should deal with gross and obvious criminality; they should not be based on the issue of whether a man can pursue a certain line of conduct and get to Heaven or into the best society socially, medically or otherwise.

---

**The Angell Memorial Hospital** was formally opened in Boston, February 25, under the auspices of the Massachusetts society for the Prevention of Cruelty to Animals, in memory of the late George Thorndike Angell, formerly president. It contains aseptic operating rooms for small animals and horses, a veterinary clinic and "wards" with cages and stalls for all kinds of stock animals. In addition to preventing suffering, it will encourage scientific veterinary practice and research.

---

**The Bicycle.** Moving pictures of the war emphasize the fact that the bicycle, once the rage in America and now quite unfashionable, remains in both economic, military and general use in Europe. At this time of year, physicians should bear in mind the availability of the bicycle for hygienic exercise, and also its utility at times when the automobile is not at hand. A case of poisoning, with the car locked up some distance away, has reminded us that, even as a practical part of the physicians equipment, the bicycle is still a live issue. The quiet, lack of vibration, possibility of getting into the country and of enjoying scenery en route, without the constant nervous tension apprehension of breakdowns and liability to dangerously strenuous physical exercise, the demand for moderate, continuous exercise by groups of muscles naturally stronger under modern conditions, give the bicycle much to balance against the greater speed and prestige of the automobile.

---

**Amendment to Sanitary Code of Buffalo Declared Unconstitutional.** The principle point involved was the right of the Health Commissioner to require the filing of formulas of patent medicines which were declared to be "the poor man's doctor." Charges of a "medical trust" were made. It was also declared that the state—not to mention the federal laws—were already sufficient. We are inclined to think that patent medicines are rather any man's poor doctor, and we take little stock in the idea of a medical trust or in the tendency to antagonize physicians and pharmacists whose relations ought to be close and cordial. Still, municipal legislation should be limited to purely local and minor issues and we do not believe in going too far in the direction of protecting anyone against his own will.

---

**The Deadly Air Hose.** Edward Devine of Albany was killed during hazing by his fellow workmen in a rail-road shop, by a blast of air at 110 pounds, directed at the anus, March 25. In spite of the wide publicity given the case in the newspapers, perhaps because of it, the same experiment was tried on Harry Zahrich at the Lackawanna Steel Plant, as an April first joke. He was cared for at the Moses Taylor Hospital.

Having witnessed the necropsy on the first case of intestinal perforation by this method in Buffalo, several years ago—if, indeed, this was not the first case from compressed air in industries—we can appreciate the tremendous damage due to this cause. Intestinal perforation from compressed air presents somewhat different factors to those of ordinary traumatic perforation or rupture from external violence, or even from rupture from liquids. The ready diffusion of a gas, produces a nearly uniform and enormous pressure upon a large length of intestine. Thus, even if rupture is not caused, the diffuse strain results in a very high grade of shock. When rupture does occur, bacteria are driven into the peritoneal cavity violently and are less readily removed than when localized infection occurs from puncture, or from the abrasion of a part of the intestinal wall against a resisting bone, by external pressure, or from the breaking of an abscess or ulcer. The extent of the lesion also increases the difficulty of the operation and prolongs and increases the distinctly surgical shock. This method of doing damage to one's fellows, is not entirely new. With less elaborate apparatus and with more frequent use of water than of air, it was an established method of torture during the dark ages. Probably none of the recent cases were deliberately homicidal. The air hose appeals to the practical joker. The site of application conforms exactly to his conception of humor. He acts quickly and without the realization that serious harm can occur. Compressed air is now so readily accessible in various industrial plants and for inflating tires, that it is a wonder that more fatal or serious cases have not occurred. Warning should be given to all employes wherever compressed air is available.

A word may be added as to the physiology of the anal sphincter. From the difficulty of introducing specula or the examining finger, the medical profession is, perhaps, unduly impressed with the strength of the protection afforded. It should be noted that, physiologically, the sphincter is called upon to resist pressure only from above. Inevitably, with psychic and mechanic stimuli from below, considerable reflex resistance occurs but this is not so much in evidence in regard to the resistance to fluids, either liquid or gaseous. In some of the traumatic air-hose cases reported, it has been distinctly stated that the air current entered through the ordinary clothing. With the body immersed in warm water, even a very moderate stream of water will enter the anus from a hose; indeed water is sometimes admitted in swimming.

---

**Venereal Prophylaxis in the Navy.** Referring to the letter to this Journal from Josephus Daniels, Secretary of the Navy, page 435, February issue, it is of interest to note a circular letter addressed by him to all commanding officers, under date of February 27. In this he properly considers the navy responsible for the morals of the young men enlisted, the majority being under 25. He speaks in a personal fatherly, and Christian way. In his eyes, the navy is not a machine but an aggregation of boys and men in whom the officers must take the same interest that their parents did before they entrusted them to others. He speaks without apology of the "foundations of our moral and Christian beliefs." He considers that to let a youth go on shore with a venereal prophylactic packet in his pocket, is a strong hint that he shall use it. During the last statistic year venereal diseases caused 4 deaths directly, 138 discharges for disability and 141,378 individual days' disability. He advises stoppage of pay during such incapacity and points out to his critics that the navy has established "probably the most careful and thorough system of prophylaxis anywhere in the world"—as we understand, applied after return from shore and admission of venereal exposure.

---

**The $600,000 Bond Issue For a Tuberculosis Hospital** for Buffalo, has finally been secured. There has been considerable opposition, both from obvious reluctance to increase the municipal indebtedness, which is well up to the legal limit and from the feeling on the part of some that the plans would not result in a material increase in the number of patients that could be treated. There is no question but that an investment of $6 per average family is a heavy charge against certain ones, in the way of compulsory philanthropy, although very reasonable as an average, and in consideration of the fact that each pair of average families may expect, under existing conditions, a death from tuberculosis, eventually. Unfortunately, modern social tendencies seem to be working toward a fairly permanent distinction of taxable and of proletariat classes, the great majority of the former being withheld from the receipt of philanthropy and yet not able to give, voluntarily or by compulsion, except at considerable sacrifice. Now that the hospital is assured, all concerned in planning and managing it, should be impressed with the need of economy and efficiency. Bearing in mind the rapid and radical changes in hospital construction indicated by past experience, it is both a matter of immediate economy and of future efficiency, to build not with the idea of establishing a permanent and imposing monument but of erecting a fairly cheap structure or series of structures which will house comfortably, as many cases as may be expected on a liberal estimate, and which may be replaced in a generation. It is human nature to flatter ourselves on our rapid advance in the immediate past but to imagine that we have nearly reached the acme of perfection, and, therefore, to establish as permanencies, what are really crude and temporary make-shifts. It is perfectly possible that the tuberculosis problem, as it exists today, will be radically altered in the comparatively near future. On the one hand, the assumption of duties by larger and more unified phases of government, continued improvements in transportation and the possible demonstration of older and, at present, somewhat unfashionable views of the value of special climates and altitudes, may almost eliminate the local care of the tuberculous. On the other hand, the demonstration of efficiency of other methods of treatment, the gradual attrition of society to a nearly uniform financial level and the gain of socialistic views, may render it necessary to treat practically all cases of tuberculosis in publicity supported institutions, and locally. And, at the risk of seeming too optimistic, we must also bear in mind the possibility that the various sanitary and hygienic measures now well begun, in connection with improved therapeutic measures, may almost eliminate the problem of tuberculosis, even within the lifetime of persons now active in the work. It is impossible to prophesy but there is every indication for building, adequately, but only for the comparatively near future.

---

**The Fly Campaign.** It will be remembered that for the last two or three years, we have campaigned against fly-swatting in the literal sense, because of the relative inefficiency and danger of this method. Especially, we have warned against enlisting the services of children in direct attacks on flies and against the method of encouraging them to accumulate dead flies for bounties. The Buffalo Sanitary Bulletin, March 31, offers sensible suggestions for a fly campaign. Early in the season, it does pay to swat or catch the individual fly, on account of the enormous procreation of this little pest. Otherwise, the methods to be adopted are: cleanliness, destruction of fomites so far as practicable; covering or screening of garbage, manure, etc., use of efficient screens for doors and windows, fly traps, of some form or other to catch flies accidentally entering houses or attracted to fomites, education to compel general co-operation against flies.

---

**Laboratory Week** will be observed by Parke, Davis & Co., June 7 to 11. Physicians are cordially invited. Complete programs will be furnished on request, the general arrangement of the work being as follows:

Introductory Address: E. M. Houghton, Ph. G., M. D.,

Director Biological and Research Laboratories

Scientific Research, the Basis of Pharmaceutical Progress

........................Dr. J. M. Francis, Chief Chemist

Immunity ........................N. S. Ferry, Ph. B., M. D.

Chemistry and Application of the Coal-Tar Disinfectants,

..............................H. C. Hamilton, M. S.

Actively Immunizing Agents .......N. S. Ferry, Ph. B., M. D.

Ozena ..............................H. C. Ward, M. S.

Serum Therapy ....................W. E. King, A. M., M. D.

Organo-Therapy ...........Carey Pratt McCord, A. B., M. D.

Spirochaetal Diseases ...........F. W. Baeslack, A. M., M. D.

Glandular Syndromes ......Carey Pratt McCord, A. B., M. D.

Anaphylaxis and Serum Sickness....N. S. Ferry, Ph. B., M. D.

The Cancer Problem ............F. W. Baeslack, A. M., M. D.

Tuberculins ..........................A. W. Lescohier, M. D.

Sero Diagnosis ..................F. W. Baeslack, A. M., M. D.

Protective Enzymes ........Carey Pratt McCord, A. B., M. D.

Autogenous Vaccines ..............N. S. Ferry, Ph. B., M. D.
The Propagation of Experimental Animals and Maintenance of Large Animals for Biological Purposes....
..............................R. H. Wilson, D. V. M.
Sero-Enzyme Tests for Pregnancy, Syphilis and Tuberculosis ...............................H. S. Ward, M. S.
Anatomical Specimens of Glands of Internal Secretion,
.......................Carey Pratt McCord, A. B., M. D.
Rat Sarcomata, Spirochaeta Pallida and Trypanosomes...
..........................F. W. Baeslack, A. M., M. D.
Filterable Viruses .................W. E. King, A. M., M. D.
Pharmacological Action of Drugs .......R. C. Hamilton, M. S.
L. W. Rowe, B. S.
Sterilization With the Ultraviolet Ray ........L. Davis, S. M.
Rabies .................................L. T. Clark, B. S.
Luetin ........................F. W. Baeslack, A. M., M. D.
The Propagation of Experimental Animals .............
.................................R. H. Wilson D. V. M.

---

**The Rockefeller Foundation,** International Health Commission, Washington, D. C., announces a change of address, April 1, 1915, to 61 Broadway, New York, City. The Rockefeller Sanitary Commission was succeeded January 1, 1915, by The International Health Commission.

---

**The Metropolitan Life Insurance Co.** has offered $1,000 for the best essay on social hygiene for adolescents of 12 to 16. The competition is closed July 31. For particulars address American School Hygiene Association, 105 West 40 St., N. Y.

---

**Physical Examination Before Prescribing.** Our editorial confrere, Dr. C. F. Taylor calls attention to Assembly bill No. 671, by Mr. Bloch, and making this requirement. Splendid requirement in poisoning, acute mania, syncope of advanced complicated cardiac disease, etc., and so easy to establish judicially just what physical examination means. As in the Boylan, Harrison, Mann and a host of other laws, we have the same underlying fallacy that, if an idea is a good one, it ought to be codified into a law and inforced without regard to circumstances. Such legislation tends to break down the protection of individual conscience, common sense and responsibility and to substitute for them a maze of arbitrary rules.

---

**Tuberculosis Hospital Referendum.** John Leo Sullivan introduced into the Assembly of N. Y., a bill empowering the supervisors of any county to submit the matter of establishing a tuberculosis hospital to the voters at any general election. The governor has signed the bill.

**Christian Scientists Not Exempt.** The Thorn bill, to exempt from the general requirements, educational, as to age, etc., applying to medical practice, has been voted down in the assembly.

---

**The Bundle of His** was first described by Stanley Kent in the Journal of Physiology, 1893, by His, Jr., in "Arbeiten aus der Med. Klinik," Leipzig, in the same year, and naturally, more in extenso.

---

**The Louis Livingston Seaman Medal** for progress and achievement in the promotion of hygiene has been awarded to Gen. Wm. C. Gorgas.

---

**The Prejudice Against the Wrist Watch** has seemed somewhat arbitrary, and analogous to similar prejudices against certain methods of wearing the hair, choice of smoking material, mounting of lenses for ocular defects, etc., and it has even seemed that the wrist watch was extremely convenient for military service. However, recent experience includes several cases of extensive laceration of soft parts, demolition of bones, etc., requiring amputation, in cases which would otherwise have been simple ones of nearly aseptic penetration. The difference is due to the driving of fragments of glass and works through the wrist.

---

**The Riberi Prize** (13th offering) of about $4,000 will be awarded by the Royal Academy of Medicine of Italy for the best work of medical research presented up to December 31, 1916. Further information may be obtained of Dr. V. Oliva, Turin, Italy.

---

**Personal Administration.** The Harrison Law, not by accident, but in consideration of arguments offered before its passage, exempts from record, drugs personally administered by a physician to a patient. But, by recent construction, the administration is personal only if the physician goes to the patient, and not if the patient goes to the physician at the latter's office. There used to be some good fiction taught in schools about the three branches of government, executive, legislative and judicial, and the proper balancing of these functions. What is the use of having any wording in a law at all if a sub-branch of the executive department can rule that personal does not mean personal or otherwise alter the perfectly plain meaning of the law? So far as we can see, it would be equally proper to "rule" that physician does not mean physician, that administration does not mean administration, that a quarter-grain limit for morphine means a quarter milligram. We suggest that legislative bodies simply pass laws by title and leave the whole matter of filling in specifications to the boards that are going to construe them.

---

**Opium and Coca Law.** For the benefit of those physicians who may have overlooked the provisions of the federal Opium and Coca Law, we repeat the following:

Each physician, dentist, or veterinary surgeon who prescribes or dispenses opium or coca leaves, their salts, derivatives, or preparations, is required,

1. To register with the Internal Revenue Collector of his District, on or before March 1, 1915; to pay a tax of $1 a year (34 cents to June 30, 1915) and obtain his Registry Number, also a supply of special Order Blanks.

2. To prepare on March 1, 1915, and keep on file an Inventory of all such drugs and preparations he has on hand at that date, which must be verified by oath not later than March 5, 1915.

3. To use the special Order Blanks for all such goods as he orders and to keep a duplicate of each order on file for at least two (2) years, accessible to official inspectors.

4. To sign all prescriptions that he writes for these drugs with his full name and his registry number together with the date as issued and the location of his office, also the name and address of the person for whom such prescription is written.

5. To keep a Record Book of all such drugs dispensed or distributed by him (at his office) showing: (a) the date when dispensed or distributed, (b) the kind of drug and quantity and (c) the name and residence of the patient.

Inasmuch as Uncle Sam is usually uncompromising in dealing with malefactors, our medical friends will serve their best interests if they take the precaution to comply with all of the legal requirements as set forth.—Jour. N. Y. State Med. Assn.

---

**The Thompson Poor Law Again.** On page 540, April issue, we commented on this law and the objection raised by the State Charities Aid Assn. against the provision requiring the overseer or superintendent of the poor, commissioner of charities to investigate before admission to an institution. The S. C. A. A. have sent a circular stating their objections, on the ground that county tuberculosis hospitals are established mainly as a matter of general protection, that the applicants are not paupers but require relief, that no disgrace should be attached to the matter of gaining entrance to an institution, that ability to pay can be determined after commitment and that the law would delay the segregation of patients with diphtheria, small pox, etc. As the bill explicitly states that "when practicable, this investigation shall be made prior to the admission of such person" any reasonable construction and precedent, would prevent either prolongation of exposure to violently contagious diseases or suffering on the part of the patient. The gist of the discussion turns on the conception of society as being obligated as a business matter to furnish relief to the disabled or as giving relief as a matter of philanthropy which is compulsorily included in the general tax rate. Without discussing the desirability of a purely fraternal and socialistic evening up of demand and supply of the various necessities and comforts of life, and the concomitant diminution of individual and familial responsibility, the fact remains that we have not as yet reached this basis. No one contributes labor, brick, cement, etc., to the erection of the various institutions under discussion nor food and fuel for their maintenance, on the theory that he may himself have a subsequent claim upon them. The medical profession, which is largely instrumental in the conduct of such institutions may donate most of its services in case of need, but it is not supported by society as a whole on the theory that its ministrations are a general necessity, free to all. Under these circumstances, we fail to see how the indigent person who applies for medical relief, because he has not been able to make the provision which the average self-supporting person finds necessary, can be considered as distinctly different from the one who applies for food or coal or clothes. This does not imply that the applicant for any of these is unworthy, or should be degraded, or should be treated with any but the greatest consideration. But we can not see why an efficient investigation of individual needs should not be made nor why the official responsible should not be the proper one to make the investigation.

The bill further provides that if the applicant, or the father mother, husband, children or grandchildren, come into the possession of real or personal property within ten years, an action may be brought to recover, from whomever has the property. While, in general, the family should be considered a unit, there are many instances in which the action of a member justifies the relatives in refusing responsibility and so broad and long an insistance upon responsibility seems unwise, if it is even within the legal rights of the state.

---

**The Hinman Bills,** four in number, have passed the Assembly and are before the Senate Public Health Committee. These bills, in various ways, curtail the activities of the State Department of Health and, while making a specious appeal to economy, are very questionable in their ultimate wisdom. Print No. 1835, requiring the Commissioner and each director of a division to devote his entire time to the duties of office, seems to us a good bill on general principles though we understand that its immediate effect would be to drive our present efficient Commissioner, from public service.

---

**Niagara's Silver Jubilee.** The class of 1890, Niagara University, held their 25th Jubilee Banquet at the Hotel Statler, Thursday evening, April 15.

Instead of confining the dinner to the '90 class, it was made a reunion of Niagara University graduates and teachers; fifty-eight were present. Dr. L. J. MacAdam, of Barkers, acted as toastmaster calling upon the Very Rev. C. M. Drennan, President of Niagara University for the opening response. Dr. Drennan enjoys the distinction of being one of the most forcible after dinner speakers in the country; his remarks, "Niagara University, its aims and results," was most gratifyingly received by the members present.

Dr. W. G. Bissell, formerly Prof. of Bacteriology, gave a glowing account of what microscopical medicine was in the early days, and what it is today. He asked that all graduates use every effort to prevent the passage of Medical Legislation at Albany detrimental to Scientific Medicine. As has been usual in the past, and there was no exception at this time, Dr. Edward M. Dooley in his characteristic way held his listeners spell-bound while in rhyme he described the men of old, as compared with those of today. Much merriment was created when he referred to one of his class-mates saying, "that although his fame is known from coast to coast, in Chicago he struck a snag: it appeared like a ghost." Dr. Geo. H. Kennedy in reply, "the Doctor," dealt with the subject in his masterly and characteristic manner, remarking that a class of men with the intelligence, forethought and moral characteristics possessed by them, more attention should be given to civic duties, for it is such teachings and examples that is one of the greatest aids in the upbuilding of the human race.

Other responses were by Drs. H. C. Buswell; L. G. Hanley; J. Henry Dowd, and the only living incorporator of Niagara University, Dr. Geo. E. Fell, the inventor of what is known today as the Pulmotor.

Beautiful half-tone souvenirs of the class of 1890 were enclosed in the menu cards.

---

**Vicarious Menstruation.** Hirschberg, Zentralblatt für Gyn., 1914, No. 26. Regular menstrual discharges were accompanied by a watery discharge from the nipples from the 15th to the 17th year, later becoming bloody. The flow ceased during two pregnancies terminated by abortion and during the third, completed at term but did not recur after this third pregnancy. Another case is reported following hysteropexy with removal of ovaries.

